# Self-reported and genetically predicted effects of coffee intake on rheumatoid arthritis: Epidemiological studies and Mendelian randomization analysis

**DOI:** 10.3389/fnut.2022.926190

**Published:** 2022-09-12

**Authors:** Bin Pu, Peng Gu, ChuRong Zheng, LiQiong Ma, XiaoHui Zheng, ZhanPeng Zeng

**Affiliations:** ^1^Guangzhou University of Chinese Medicine, Guangzhou, China; ^2^The First Affiliated Hospital, Guangzhou University of Chinese Medicine, Guangzhou, China

**Keywords:** coffee intake, rheumatoid arthritis, national health and nutrition examination survey, Mendelian randomization, epidemiological study

## Abstract

**Background and aims:**

Causal research concerning coffee intake and rheumatoid arthritis (RA) risk is controversial. The objective of this study was to further explore the causal relationship between coffee intake and RA risk.

**Methods:**

The 4,310 participants from NHANES 2003–2006 were included in an epidemiological study to assess the association between coffee intake and RA by weighted multivariate logistic regression. The inverse variance weighted (IVW) method of two-sample Mendelian randomization (MR), employing genetic data from UK Biobank (428,860 cases) of coffee intake and MR-Base platform (14,361 cases and 43,923 controls) of RA, was performed to estimate the causal relationship between coffee intake and RA.

**Results:**

Weighted multivariate logistic regression suggested no significant correlation between coffee intake and RA. Compared to the no-coffee group, the odds ratio for RA in the <1, 1–3, ≥4 cups/day group were 1.297, 1.378, and 1.125 (*P* = 0.204, 0.098, and 0.698, respectively). In the IVW of MR analysis, there was no causal relationship between coffee intake and RA (OR = 1.47, *P* = 0.218).

**Conclusion:**

Our study did not support a causal association between coffee intake and RA risk. However, it is necessary to consider valid information on coffee intake, including brewing method, type of coffee, and quantity, in further analysis of coffee intake and RA.

## Introduction

Rheumatoid arthritis (RA) is a common chronic rheumatoid immune disease characterized by synovitis, cartilage, and symmetrical joint damage (1). RA can affect individuals of any age and its prevalence rate is estimated at 0.5–1% worldwide (2). The pathogenic factors of RA are unclear, which may be related to infection, hormone secretion disorder, genetics, etc. (3, 4). Coffee is one of the most popular beverages in the world, with about 85% of Americans reportedly consuming at least one caffeinated beverage per day (5). Because of the widespread popularity and easy availability of coffee, the public and the scientific community are extraordinarily interested in the relationship and interaction between coffee and essential health. Coffee contains hundreds of bioactive compounds, including caffeine, caffeic acid, chlorogenic acid, etc. (6).

At present, a large number of studies have indicated that coffee can affect the risk of some chronic diseases such as depression, type 2 diabetes, Parkinson's disease (7–9), and RA. Among them, we are very interested in the relationship between coffee intake and RA risk. Previous prospective studies have shown that coffee intake was not associated with RA risk (10–12). However, Mikuls et al. found that decaffeinated coffee intake was independently positively correlated with RA risk, while the caffeinated coffee intake was not associated with RA rise (13) and similar results were obtained by a meta-analysis by Asoudeh et al. (14). Overall, their results were not consistent.

Mendelian randomization (MR) is considered a method comparable to the randomized controlled study (15), which uses genetic variation as an instrumental variable (IV) to deduce the causality between the outcome and exposure. Genetic variation is randomly assigned at conception to effectively avoid the confounding bias and reverse causal bias of traditional epidemiological studies (16). Moreover, as the effect of genetic variation is much longer than clinical intervention, the actual causal effect estimated by MR is more significant and more accurate than the clinically observed causal effect, including the meta-analysis of clinical research and prospective studies (17).

Therefore, this study intended to use the extensive database of the National Health and Nutrition Examination Survey (NHANES) to conduct a cross-sectional study on the correlation between coffee intake and RA risk, followed by the MR study to evaluate the causal relationship between coffee intake and RA from the level of genetic variation.

## Materials and methods

### Observational epidemiological analysis

NHANES is a series of continuous cross-sectional surveys conducted jointly by the National Center For Health Statistics and the Centers for Disease Control And Prevention. We collected two consecutive NHANES two-year cycles (2003–2006) in total since participants responded to the question “Did you drink coffee” in the two cycles (18). The individuals, under 20 years old (*n* = 11,650), with missing coffee intake data (*n* = 3,283), unknown diagnosis of arthritis (*n* = 670), and missing data of related covariates (*n* = 1,757), were excluded (Supplementary Figure 1). Finally, a total of 4,310 individuals were included in the final analysis. Detailed data is accessible on https://wwwn.cdc.gov/nchs/nhanes/Default.aspx.

### Variables included in observational epidemiological analysis

RA was defined based on the items in the medical conditions questionnaire: “Doctor ever said you had arthritis?” and “Which type of arthritis?”. Respondents who responded only RA were classified as RA. According to the question, “How many cups of coffee, caffeinated or decaffeinated, did you drink?”, we divided coffee intake into four groups: no consumption, <1, 1–3, and ≥4 cups/day. According to the previous study, we used the following variables as covariates: sex, age, race, education level, Body mass index (BMI), smoking status, poverty-income ratio, alcohol, high-density lipoprotein, total cholesterol, hypertension, and diabetes condition (19, 20). In addition, through screening, total calcium, phosphorus, kidney disease, and CRP may also be associated with RA risk (21–23), so they were also included in the covariates.

### Sources of two-sample MR

UK Biobank is a large-scale biomedical database and research resource containing in-depth genetic and health information from 500,000 British participants. The genome-wide association study (GWAS) summary data set for coffee intake (ukb-b-5237) based on UK Biobank includes over 428,860 samples of European ancestry. Detailed information regarding the phenotype and quality control process in UK Biobank is available on the website (https://www.ukbiobank.ac.uk/). We also collected the aggregated data on RA (ieu-a-832) from a previous study containing 582,84 participants of European ancestries. All RA cases in this study fulfilled the 1,987 criteria of the American College of Rheumatology for RA diagnosis or were diagnosed as RA by a professional rheumatologist (24).

### Statistical analysis

In the observational epidemiological analysis, the baseline characteristics of all study participants were described by average (continuous variables) or proportions (classified variables). We executed weighted multivariate-adjusted logistic regression to calculate the odds ratio (OR), *P*-value, and 95% confidence interval (CI) between different coffee intakes and RA risk.

MR is a method that accomplishes genetic variation to estimate the causality between exposure and outcome. MR's validity is based on three hypotheses: (1) the genetic IVs are related to the exposure factors, (2) IVs are independent of confounders, and (3) IVs act only through exposure factors to outcomes (25) (Supplementary Figure 2). To construct genetic instruments for coffee intake and RA, we obtained the SNPs (single nucleotide polymorphisms) that are reliable (*P* <5 × 10^−8^) and independent (*r*^2^ <0.001, kb = 10,000) of coffee intake. We chose not to use the SNP proxy and set the minimum allele frequency (MAF) to 0.01. In addition, we coordinated the effect alleles in the exposure and result data sets, excluding all SNPs with palindromes. The fixed-effect IVW, MR-Egger, weighted median, weighted mode, and simple model were used to examine a causal association and IVW was considered the primary analytical method (26). The MR-Egger tested its horizontal pleiotropic. When the value of the intercept term was close to 0 and *P* > 0.05, it indicated that there was no horizontal multiplicity (27). If horizontal multiplicity exists, the SNP with horizontal multiplicity was removed with CAUSE (Causal Analysis Using Summary Effect estimates) packages. The IVW and MR-Egger were used to quantify the heterogeneity effect between the genetic instruments and heterogeneous SNP was removed using the MR-PRESSO (Mendelian Randomization Pleiotropy RESidual Sum and Outlier) packages. F-statistics were used to test weak instrumental variables. *R*^2^ is the variance of coffee intake explained by genetic instruments, *K* is the number of genetic variants (Because we calculated the F value of each SNP, which was *K* = 1), and *N* is the sample size (28). *F* > 10 indicates that weak IV deviations are unlikely (29).

*R*^2^ was calculated as follows:


$$R^{2}=2\times (1-EAF)\times EAF\times {\left(\frac{BETA}{SE\times \sqrt{N}}\right)}^{2}$$

The F-statistic for each SNP was calculated as follows:


$$\begin{array}{l} F=\frac{N-k-1}{k}\times \frac{R^{2}}{1-R^{2}} \end{array}$$

In addition, the leave-one-out method was used to deal with sensitivity analysis, in which the SNP was removed one by one; the estimated value of the remaining IVs was calculated and compared with the total estimate to test the impact of each SNP (30).

All data were analyzed by packages “TwoSampleMR,” “MR-PRESSO”, and “CAUSE” in R software v.4.0.3, and EmpowerStats software. All images were generated by GraphPad Prism 9.0.0 and Adobe Illustrator 2021. *P* < 0.05 indicated statistical significance.

## Results

### Epidemiological observation and analysis

Participants' information was extracted from the NHANES 2003–2006. After inclusion and exclusion criteria screening, we recruited 4,310 participants in the final analysis (Supplementary Figure 1). Table 1 compares the baseline characteristics of participants with RA and without RA in the study sample. The average age of 275 participants with RA was 60.6 years (45.3–75.8 years), and 4,035 participants without RA was 49.0 years (30.4–67.8 years). The prevalence rate of RA was 6.4%.

**Table 1:** Baseline characteristics of study participants with or without rheumatoid arthritis in the NHANES 2003–2006.

**Characteristics**	**Total number of participants (*n =* 4,310)**		***P***
	**RA (*n =* 275)**	**Non-RA (*n =* 4,035)**	
Age (years, mean ± SD)	60.64 ± 15.14	49.04 ± 18.69	<0.001
Race/ethnicity, *n* (%)			0.018
Mexican American	46 (16.73%)	775 (19.21%)	
Other Hispanic	6 (2.18%)	105 (2.60%)	
Non-Hispanic White	136 (49.45%)	2,264 (56.11%)	
Non-Hispanic Black	76 (27.64%)	725 (17.97%)	
Other Race	11 (4.00%)	166 (4.11%)	
Education, *n* (%)			<0.001
Under high school	101 (36.73%)	946 (23.44%)	
High school or equivalent	79 (28.73%)	976 (24.19%)	
Some College or AA degree	69 (25.09%)	1,176 (29.14%)	
College Graduate or above	26 (9.45%)	937 (23.22%)	
BMI, mean ± SD (kg/m^2^)	29.86 ± 6.70	28.52 ± 6.61	0.052
Smoking, *n* (%)			<0.001
Never	76 (27.64%)	912 (22.60%)	
Former	94 (34.18%)	1,057 (26.20%)	
Current	105 (38.18%)	2,066 (51.20%)	
Had at least 12 alcohol drinks past 1 year? *n* (%)			0.068
Yes	175 (63.64%)	2,822 (69.94%)	
No	100 (36.36%)	1,213 (30.06%)	
Marital status, *n* (%)			0.477
Live with someone	161 (58.55%)	2,628 (65.13%)	
Live alone	114 (41.45%)	1,407 (34.87%)	
PIR, *n* (%)			<0.001
<1.3	82 (29.82%)	977 (24.21%)	
1.3-3.5	129 (46.91%)	1,588 (39.36%)	
>3.5	64 (23.27%)	1,470 (36.43%)	
Hypertension, *n* (%)			<0.001
Yes	157 (57.09%)	1,311 (32.49%)	
No	118 (42.91%)	2,724 (67.51%)	
Diabetes, *n* (%)			0.008
Yes	49 (17.82%)	374 (9.27%)	
No	216 (78.55%)	3,617 (89.64%)	
Borderline	10 (3.64%)	44 (1.09%)	
Kidney disease, *n* (%)			0.007
Yes	18 (6.55%)	84 (2.08%)	
No	257 (93.45%)	3,951 (97.92%)	
Phosphorus (mmol/L, mean ± SD)	3.74 ± 0.50	3.81 ± 0.54	0.772
Total calcium (mmol/L, mean ± SD)	9.46 ± 0.41	9.51 ± 0.37	0.021
CRP (mmol/L, mean ± SD)	0.68 ± 1.19	0.45 ± 0.83	0.006
HDL-C (mmol/L, mean ± SD)	53.15 ± 16.22	55.22 ± 16.47	0.061
TC (mmol/L, mean ± SD)	196.63 ± 43.67	202.06 ± 43.16	0.767
Coffee intake, *n* (%)			0.057
None	45 (16.36%)	1,037 (25.70%)	
<1 cup/day	70 (25.45%)	1,086 (26.91%)	
1–3 cups/day	141 (51.27%)	1,638 (40.59%)	
≥4 cups/day	19 (6.91%)	274 (6.79%)	

NHANES, National Health and Nutrition Examination Survey; SD, standard deviation; Values are mean ± SD or n (%); HDL-C, high-density lipoprotein cholesterol; BMI, Body mass index; CRP, C-reactive protein; TC, total Cholesterol; PIR, Poverty Impact Ratio; CI, confidence interval; β, regression coefficient.

Supplementary Table 1 indicates the association between different coffee intakes and RA risk. Only 1-3 cups/day of coffee intake was statistically significant (adjusted for age, sex and race; OR = 1.479, 95%CI:1.027–2.130, *P* = 0.036). However, Table 2 shows no association between coffee intake and RA in the overall study population after adjustment for all variables.

**Table 2:** Weighted multivariable-adjusted a logistic regression of RA risk across coffee intake categories in NHANES 2003–2006.

**Coffee intake(cup/day)**	**OR**	**95%CI**		***P*-value**
		**Lower limit**	**Upper limit**	
0	Ref	—	—	—
<1	1.198	0.783	1.834	0.405
1–3	1.417	0.951	2.111	0.086
>3	1.026	0.539	1.950	0.938
P for trend	—	—	—	0.260
Increase per cup	1.100	0.932	1.298	—

Age, sex, race/ethnicity, Education, Smoking, Had at least 12 alcohol drinks past one year? Marital status, PIR, Hypertension, Diabetes, BMI (obese, overweight, normal), TC (quartile groups), HDL(quartile groups), Ca (quartile groups), P (quartile groups), CRP (quartile groups) were adjusted in the model.

### The results of the two-sample MR analysis

**Figure 1:**
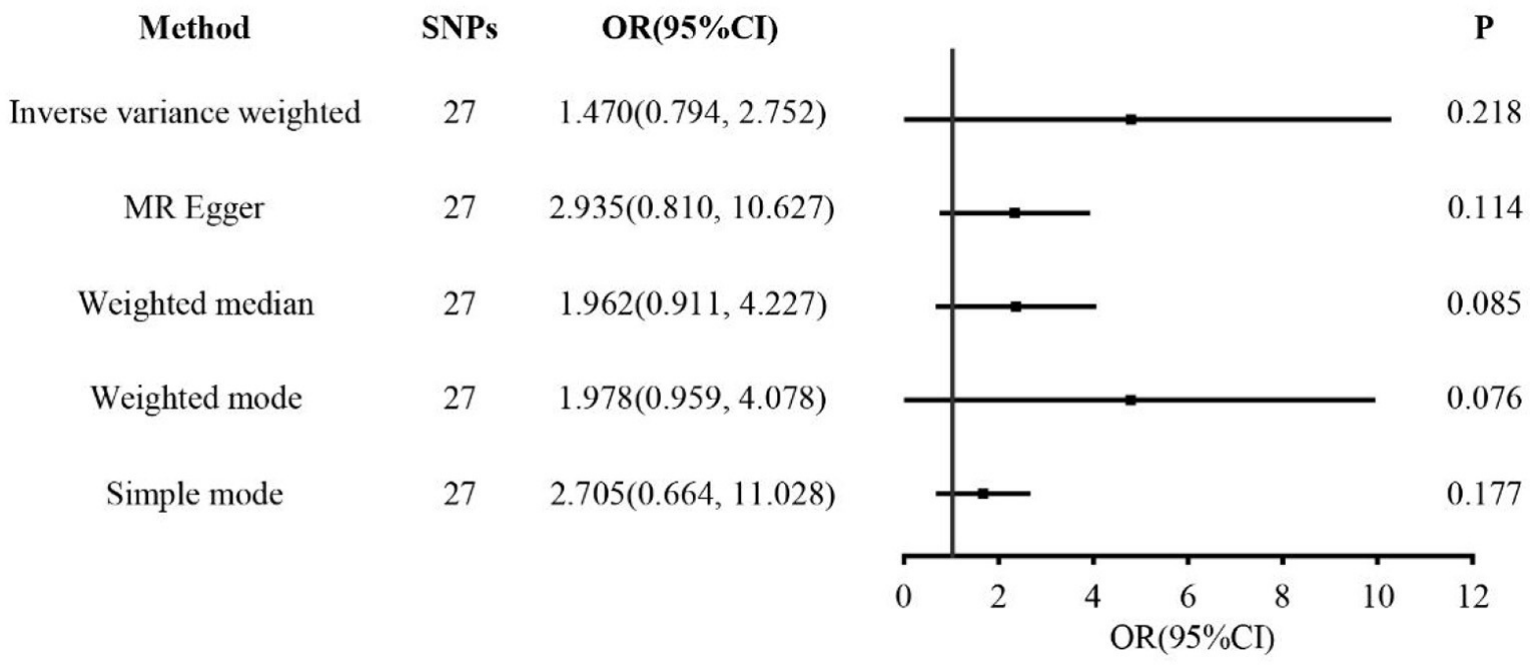
Forest plots of MR study using genetically predicted coffee intake with RA. IVW, MR-Egger, weighted median, weighted mode, and simple mode were used in this study.

**Figure 2:**
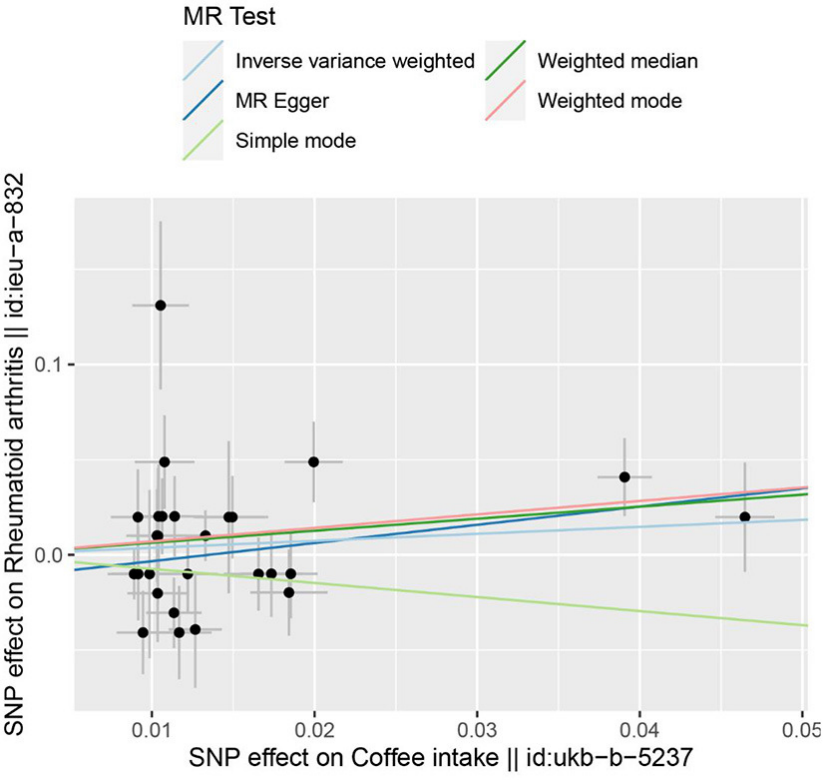
The scatter plot for MR analyses of causal associations between each coffee intake SNP and RA.

In the two-sample MR analysis, 27 SNPs related to coffee intake were selected (Supplementary Figure 3) (through linkage disequilibrium analysis, *P* < 5 × 10^−8^, *r*^2^=0.001, Kb=10,000). The F statistics of each SNP was >10 (Table 3), and there was no significant correlation with outcome variables, indicating an absence of weak instrument bias. The fixed effect IVW results showed that coffee intake had no significant effect on RA risk (*P* = 0.218); similar results were observed in MR-Egger regression, weighted median, weighted mode, and simple mode (Figure 1). The scatter plot of these results is shown in Figure 2. No evidence of horizontal pleiotropic in the MR-Egger regression was detected (regression intercept = −0.013, *P* = 0.245). The *P*-values of MR-Egger and IVW methods were 0.059 and 0.049, respectively, indicating no significant heterogeneity between IVs and visualization results indicated in the funnel plot (Supplementary Figure 4). Leave-one-out sensitivity analysis showed that the comprehensive effects of removing any SNP were unchanged or reversed, indicating that the results were credible (Supplementary Figure 5). The forest plots of coffee-RA estimates in each SNP are presented in Supplementary Figure 6.

**Table 3:** Characteristics of SNPs associated with coffee consumption.

**SNP**	**EA**	**EAF**	**BETA**	**SE**	**P1**	**P2**	***N***	***R* ^2^**	***F***
rs1057868	T	0.28	0.0200	0.0018	5.40E−29	0.0210	428860	0.0001187	51
rs12514566	A	0.34	−0.0114	0.0017	2.40E−11	0.3400	428860	0.0000465	20
rs12989746	T	0.25	0.0104	0.0019	2.80E−08	0.4300	428860	0.0000269	12
rs13054099	C	0.26	−0.0108	0.0018	4.30E−09	0.0470	428860	0.0000310	13
rs13163336	A	0.16	0.0149	0.0022	1.30E−11	0.3600	428860	0.0000283	12
rs1338549	G	0.53	−0.0095	0.0016	5.60E−09	0.0620	428860	0.0000394	17
rs13387939	A	0.83	0.0166	0.0021	9.80E−15	0.6000	428860	0.0000397	17
rs1421085	C	0.40	0.0185	0.0016	1.70E−29	0.6700	428860	0.0001427	61
^*^rs1527961	C	0.13	−0.0133	0.0024	1.70E−08	0.0650	428860	0.0000173	7
^*^rs17842490	G	0.01	−0.0452	0.0068	3.30E−11	0.5800	428860	0.0000029	1
rs1942965	C	0.50	−0.0089	0.0016	3.80E−08	0.4500	428860	0.0000352	15
rs2465037	A	0.34	−0.0106	0.0017	4.80E−10	0.3100	428860	0.0000407	17
rs2472297	T	0.26	0.0465	0.0018	1.10E−142	0.4900	428860	0.0005844	251
rs2597805	T	0.68	0.0099	0.0018	2.00E−08	0.8200	428860	0.0000318	14
rs34060476	G	0.13	0.0184	0.0024	7.50E−15	0.3800	428860	0.0000327	14
rs4410790	C	0.63	0.0391	0.0017	1.20E−120	0.0460	428860	0.0005916	254
rs4615895	A	0.74	0.0122	0.0018	4.20E−11	0.6200	428860	0.0000390	17
rs476828	C	0.24	0.0173	0.0019	5.60E−20	0.6600	428860	0.0000707	30
rs516636	A	0.21	0.0117	0.0020	4.00E−09	0.0950	428860	0.0000267	11
rs56113850	C	0.58	0.0127	0.0016	8.90E−15	0.2000	428860	0.0000684	29
^*^rs57918684	A	0.15	0.0129	0.0022	8.60E−09	0.2900	428860	0.0000202	9
rs6062682	T	0.46	0.0104	0.0016	2.50E−10	0.7800	428860	0.0000464	20
rs6063085	C	0.37	0.0104	0.0017	4.50E−10	0.4600	428860	0.0000424	18
rs61928609	C	0.84	−0.0147	0.0022	1.30E−11	0.6200	428860	0.0000294	13
rs62064918	T	0.24	−0.0103	0.0019	4.10E−08	0.6800	428860	0.0000259	11
rs630194	C	0.34	−0.0114	0.0017	2.30E−11	0.1000	428860	0.0000470	20
rs6469262	C	0.56	−0.0092	0.0016	1.90E−08	0.6800	428860	0.0000362	16
rs780093	C	0.62	0.0133	0.0017	1.00E−15	0.4500	428860	0.0000710	30
rs7811609	T	0.37	0.0091	0.0017	4.00E−08	0.4300	428860	0.0000329	14
rs8056750	T	0.36	0.0105	0.0017	1.30E−09	0.0030	428860	0.0000395	17

SNP, singlE–nucleotide polymorphism; EAF, effect allele frequency; EA, effect allele; BETA, beta.exposure; SE, standard error; P1, pval.coffee; P2, pval. rheumatoid arthritis; R2 was calculated as follows:$R^{2}=2\times (1-EAF)\times EAF\times {\left(\frac{BETA}{SE\times \sqrt{N}}\right)}^{2}$. The F-statistic for each SNP was calculated as follows:$F=\frac{N-k-1}{k}\times \frac{R^{2}}{1-R^{2}}$.

* Three SNPs (rs17842490, rs1527961, rs57918684) being weak instrumental variables were removed, and 27 SNPs were included for MR analyses.

## Discussion

This present study used epidemiological analysis and two-sample MR analysis to explore the causality between coffee intake and RA risk. Our results did not support a causal association between coffee intake and RA risk.

RA is a chronic autoimmune inflammatory disease. Based on susceptible genes, RA initiates T cell activation and autoimmune reaction by infection, smoking, etc. A large number of inflammatory cytokines, autoantibodies, and oxygen free radicals increase, resulting in the inflammatory injury of joint tissues, synovial hyperplasia, and structural destruction of bone and cartilage (31). Firstly, its occurrence and development are closely related to inflammation. A prospective cohort study of RA in older women showed that caffeine-free coffee intake was independently positive correlated with RA incidence (13). Findings from observational studies further showed that coffee intake was associated with an increased risk of RA. Possible mechanisms include promoting the production of rheumatoid factor (32), antagonizing adenosine receptors, and inhibiting cytokine anti-inflammation (33, 34). Furthermore, Oxidative stress can lead to inflammatory infiltration of neutrophils, and free radicals can be used as oxidants and inflammatory mediators to participate in RA pathological process. Yashin et al. found that coffee contains unique antioxidants such as polyphenols, caffeine alcohol, and melanoid, which play an indispensable role in scavenging free radicals, inducing DNA repair, and activating detoxification enzymes (35–37). Secondly, immune regulation is also a vital influencing factor of RA. Sharif et al. suggested that coffee intake may have a protective effect on autoimmune diseases (38). Oxidative stress can decrease the number of activated peripheral blood mononuclear cells (PBMCs) and down-regulate immune function. The antioxidant activity of caffeic acid and its derivatives can maintain an adequate amount of PBMCs and contribute to maintaining immune function (39). Park et al. verified this theory through experiments on the effects of caffeic acid phenethyl ester on the immune system of mice (40). On the contrary, many animal experiments have proved that caffeine intake will inhibit the proliferation of B cells and T cells (41), affect antibody production and destroy the body's immune function (42).

In addition, a large cross-sectional study in South Korea reported that coffee consumption was not related to the prevalence of RA among Koreans (20). This result was consistent with our NHANES cross-sectional study. We speculate that coffee is a complex mixture, and the biological mechanisms by which it affects disease are multifactorial. On the one hand, caffeine in coffee can antagonize adenosine receptors and inhibit the production of cytokines, thus inhibiting anti-inflammatory effects (33, 34). However, antioxidants such as polyphenols and cafestol in coffee can inhibit the oxidation of the body and produce anti-inflammatory effects (35). On the other hand, the antioxidant activity of caffeic acid and its derivatives can maintain the effective amount of PBMCs and help maintain the body's immune function (39). Conversely, caffeine intake will inhibit the proliferation of T cells and B cells and destroy the body's immune function (41). In conclusion, due to the diversity of the pathogenesis of RA affected by coffee intake, the effects of multiple mediators (positive and negative) may be offset, resulting in coffee intake not being associated with RA. At present, the relationship between coffee intake and RA risk remains controversial. The main reason for this dispute may be that prospective and cross-sectional studies are easily affected by confounding factors such as environment and selection bias, so deterministic causality can not be obtained.

MR has been a popular and effective method for causal inference in recent years. It uses genetic variation as an IV to deduce the causality between outcome and exposure, which can effectively avoid the confounding bias of traditional epidemiological studies (43). Consulting the literature, we found only one MR research report on coffee consumption and RA risk—the result showed no causal relationship between coffee consumption and RA (44). However, only four SNPs in the study explained a small part of the difference in coffee consumption. A weak IV may bias the causal estimation of two-sample MR toward zero. This study selected the GWAS data set with the most prominent coffee intake and RA samples to solve the bias problem and finally screened 27 SNPs. The IVW, MR-Egger, weighted median, weighted mode, and simple mode were used to analyze the causality between the two samples. The results still indicated no causal relationship between coffee intake and RA risk. We speculate that people are an organic whole with a steady-state solid regulatory system, and the effect of coffee intake on inflammation and immunomodulatory function is not enough to cause damage to human tissues and organs. Therefore, coffee intake is unlikely to have a causality with the risk of RA.

The main advantages of this study include a large cross-sectional study based on NHANES and a two-sample MR analysis study. Cross-sectional studies can explore the relationship between coffee consumption and RA risk from the real-world epidemiological level based on population self-question-answering. MR overcomes the inherent effects of residual confusion, reverse causal deviation, and measurement errors in traditional epidemiological studies. The second advantage is that compared with previous MR studies on coffee and RA, we screened more SNPs and reduced the causal estimation bias caused by weak SNPs. However, this study has some inevitable limitations. Firstly, the cross-sectional study may have self-reported recall bias on RA diagnosis and coffee intake. Second, the results did not apply to other populations due to deviations from the data limited to American and European populations. Finally, valid information on coffee intake, including brewing method, type of coffee, and quantity, is very significant in exploring the causality between coffee intake and exposure genes, so it is necessary to consider these factors in further analysis of coffee and RA.

## Conclusions

Our study did not support a causal association between coffee intake and RA risk. However, it is necessary to consider valid information on coffee intake, including brewing method, type of coffee, and quantity, in further analysis of coffee intake and RA.

## Data availability statement

All National Health and Nutrition Examination Survey files are available from the American Centers for Disease Control and Prevention database (URL https://wwwn.cdc.gov/nchs/nhanes/Default.aspx, accessed on 6 March 2022). All the Mendelian randomization study files are available from GWAS (URL https://gwas.mrcieu.ac.uk, accessed on 17 March 2022). For all the original data, please see the Supplementary material Data Sheet 2.ZIP.

## Ethics statement

The studies involving human participants were reviewed and approved by the National Center for Health Statistics Research Ethics Review Board. The patients/participants provided their written informed consent to participate in this study. Written informed consent was obtained from the individual(s) for the publication of any potentially identifiable images or data included in this article.

## Author contributions

BP and PG designed the study, wrote, reviewed and edited the manuscript. CZ and LM analyzed data. ZZ and XZ reviewed and edited the manuscript. ZZ is the guarantor of this work. All authors approved the final version of the manuscript to be published.

## Conflict of interest

The authors declare that the research was conducted in the absence of any commercial or financial relationships that could be construed as a potential conflict of interest.

## Publisher's note

All claims expressed in this article are solely those of the authors and do not necessarily represent those of their affiliated organizations, or those of the publisher, the editors and the reviewers. Any product that may be evaluated in this article, or claim that may be made by its manufacturer, is not guaranteed or endorsed by the publisher.

## Abbreviations

MR: Mendelian randomization
RA: rheumatoid arthritis
NHANES: National Health and Nutrition Examination Survey
IVW: inverse variance weighted
IV: instrumental variable
GWAS: genome-wide association study
SNP
single nucleotide polymorphism
PBMCs: peripheral blood mononuclear cells
OR: odds ratio
HDL-C: high-density lipoprotein cholesterol
BMI: Body mass index
CRP: C-reactive protein
TC: Cholesterol
PIR: Poverty Impact Ratio
CI: confidence interval
β regression coefficient
SD: standard deviation
EAF: effect allele frequency
EA: effect allele
BETA: beta. exposure
SE: standard error.
