# Supplementary material for: Self-reported and genetically predicted effects of coffee intake on rheumatoid arthritis: Epidemiological studies and Mendelian randomization analysis
Source: Front Nutr. 2022 Sep 12;9:926190. doi: 10.3389/fnut.2022.926190 (PMC9510978; doi:10.3389/fnut.2022.926190)

Supplementary Materials

SUPPLEMENTARY NOTES

Supplementary Table1. Weighted multivariable-adjusted a logistic regression of RA risk across coffee intake categories in NHANES 2003-2006.

Supplementary Figure1. Flow chart of participants' selection.

Supplementary Figure2. Schematic Diagram of the Mendelian Randomization Assumptions.

Supplementary Figure3. Flow chart of Mendelian randomization study.

Supplementary Figure4. The funnel plot for MR analyses of causal associations between each coffee intake SNP and RA.

Supplementary Figure5. Leave-one-out sensitivity analysis for RA using SNP associated coffee intake.

Supplementary Figure6. The forest plot for MR analyses of causal associations between each coffee intake SNP and RA.

Supplementary Table1. Weighted multivariable-adjusted a logistic regression of RA risk across coffee intake categories in NHANES 2003-2006.

Coffee intake	Model1		Model2		Model3	
	OR (95%CI)	P	OR (95%CI)	P	OR (95%CI)	P
None	1.0 (—)	—	1.0 (—)	—	1.0 (—)	—
<1 cup/day	1.485(1.012,2.181)	0.043	1.383 (0.934, 2.047)	0.105	1.198 (0.783, 1.834)	0.405
1-3 cups/day	1.984(1.406,2.799)	0.000	1.479 (1.027, 2.130)	0.036	1.417 (0.951, 2.111)	0.086
≥4 cups/day	1.598(0.920,2.777)	0.096	1.369 (0.774, 2.424)	0.281	1.026 (0.539, 1.950)	0.938
P for trend	< 0.001		0.077		0.260	
Increase per cup	1.276 (1.114, 1.461)		1.144 (0.986, 1.328)		1.100 (0.932, 1.298)	

Model1, no covariates were adjusted.
Model2, age, sex, and race/ethnicity, were adjusted.
Model3, age, sex, race/ethnicity, Education, Smoking, Had at least 12 alcohol drinks past one year? Marital status, PIR, Hypertension, Diabetes, BMI (obese, overweight, normal), TC (quartile groups),HDL(quartile groups), Ca (quartile groups), P (quartile groups), and CRP (quartile groups) were adjusted in the model.

Supplementary Figure1. Flow chart of participants' selection. NHANES; Coffee Intake; Rheumatoid Arthritis.

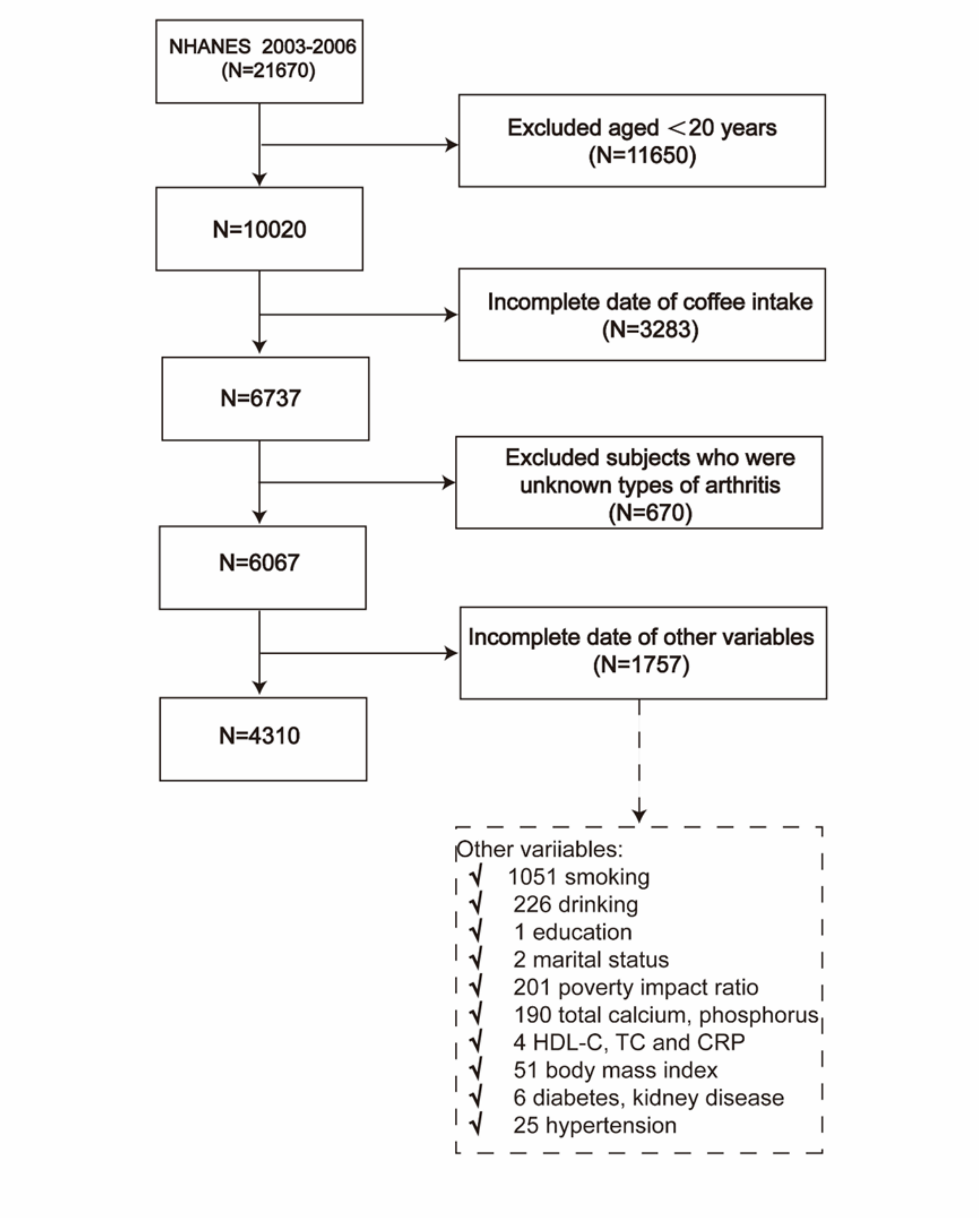

Supplementary Figure2. Schematic Diagram of the Mendelian Randomization Assumptions.

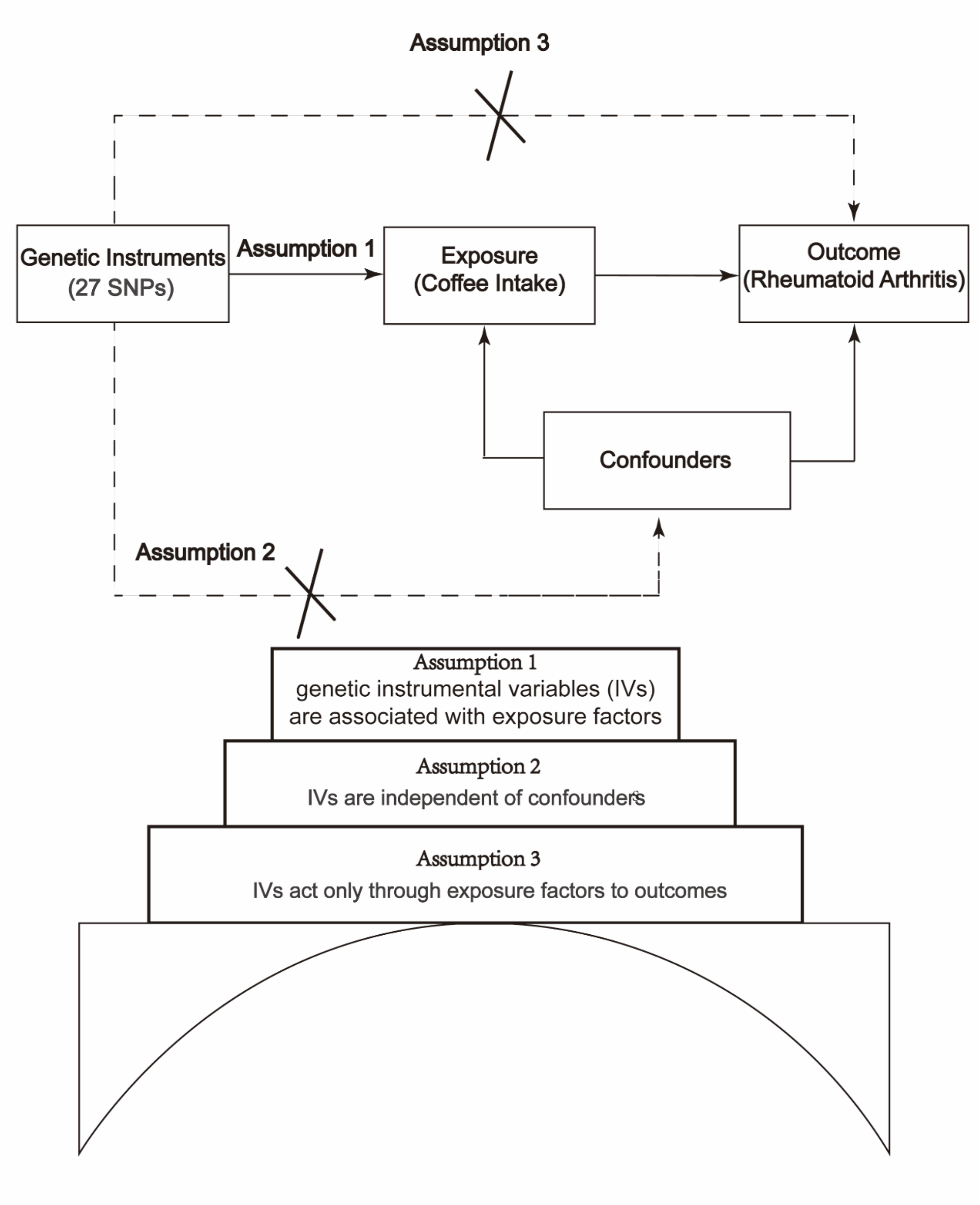

Supplementary Figure3. Flow chart of Mendelian randomization study.

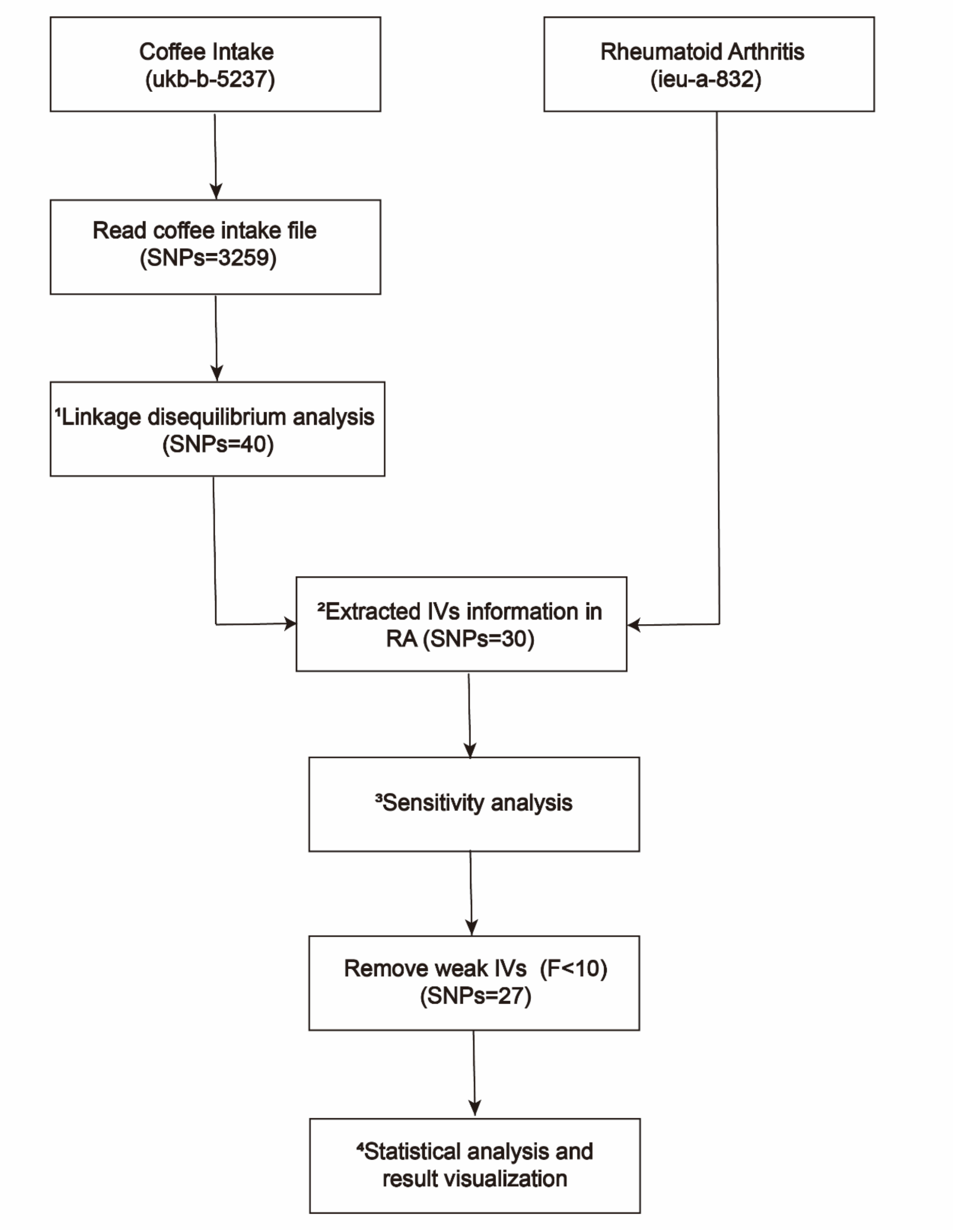

[1]Remove linkage disequilibrium: n=40, $P<5\times10\text{-}8$, $r^2=0.0001$, Kb=10000.

[2] Set the minimum allele frequency (MAF) to 0.01without using the SNP proxy and remove all SNPs with palindromes.

[3] Conducted heterogeneity test, pleiotropy test, and Leave-one-out sensitivity test.

[4] Using Inverse variance weighted, MR Egger, Weighted median, Weighted mode, and Simple mode. Drawing the scatter plot, the forest plot, the Leave-one-out sensitivity analysis plot, and the funnel plot.

Supplementary Figure4. The funnel plot for MR analyses of causal associations between each coffee intake SNP and RA.

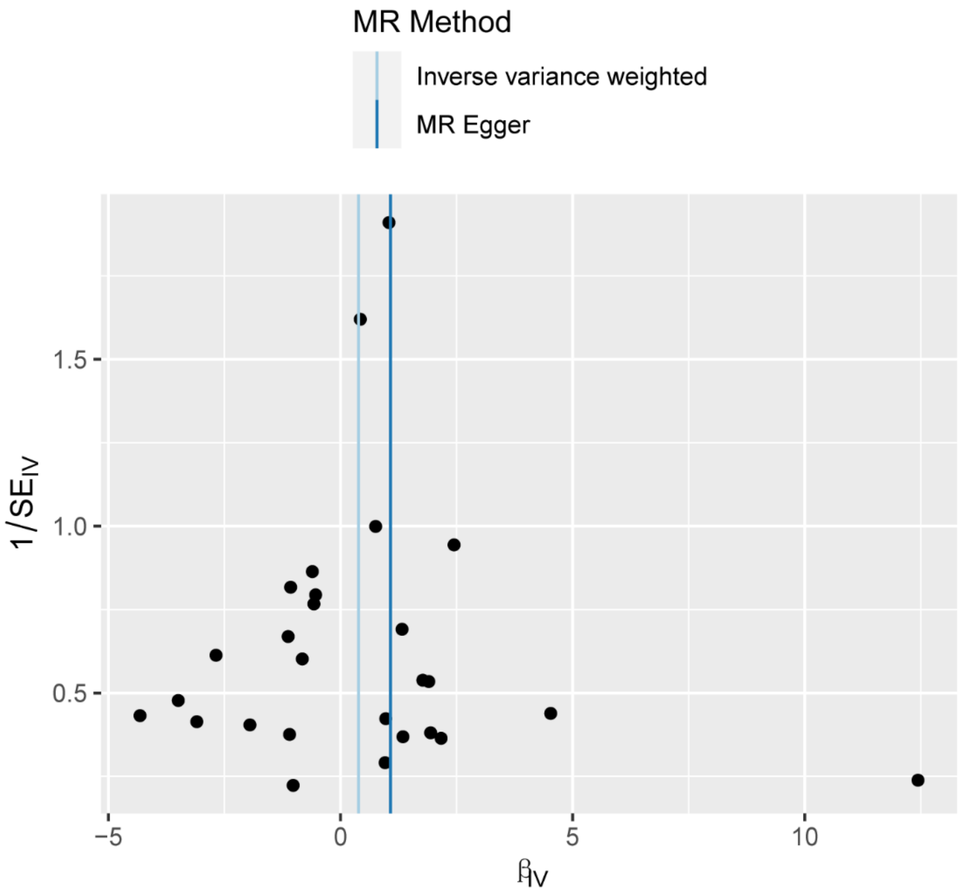

Supplementary Figure5. Leave-one-out sensitivity analysis for RA using SNP associated coffee intake.

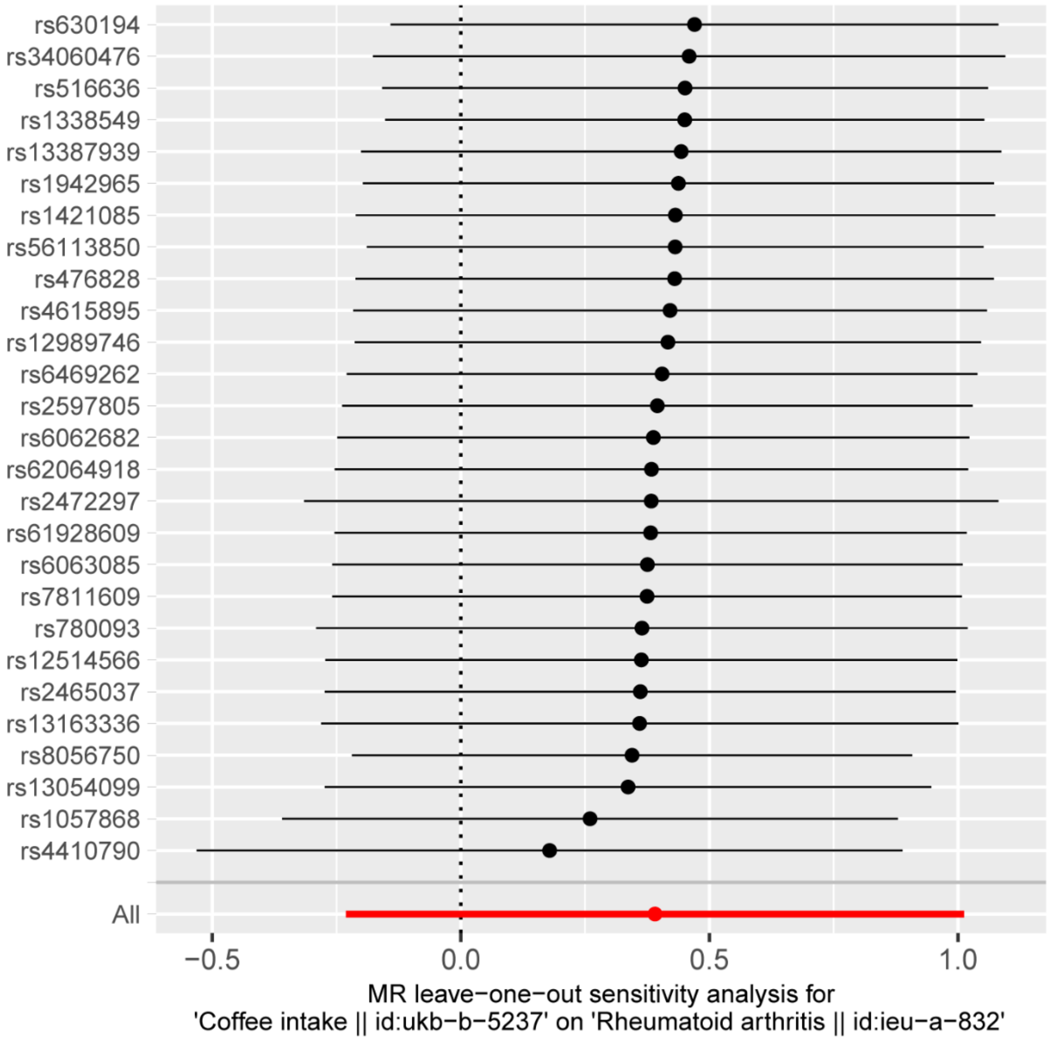

Supplementary Figure6. The forest plot for MR analyses of causal associations between each coffee intake SNP and RA.

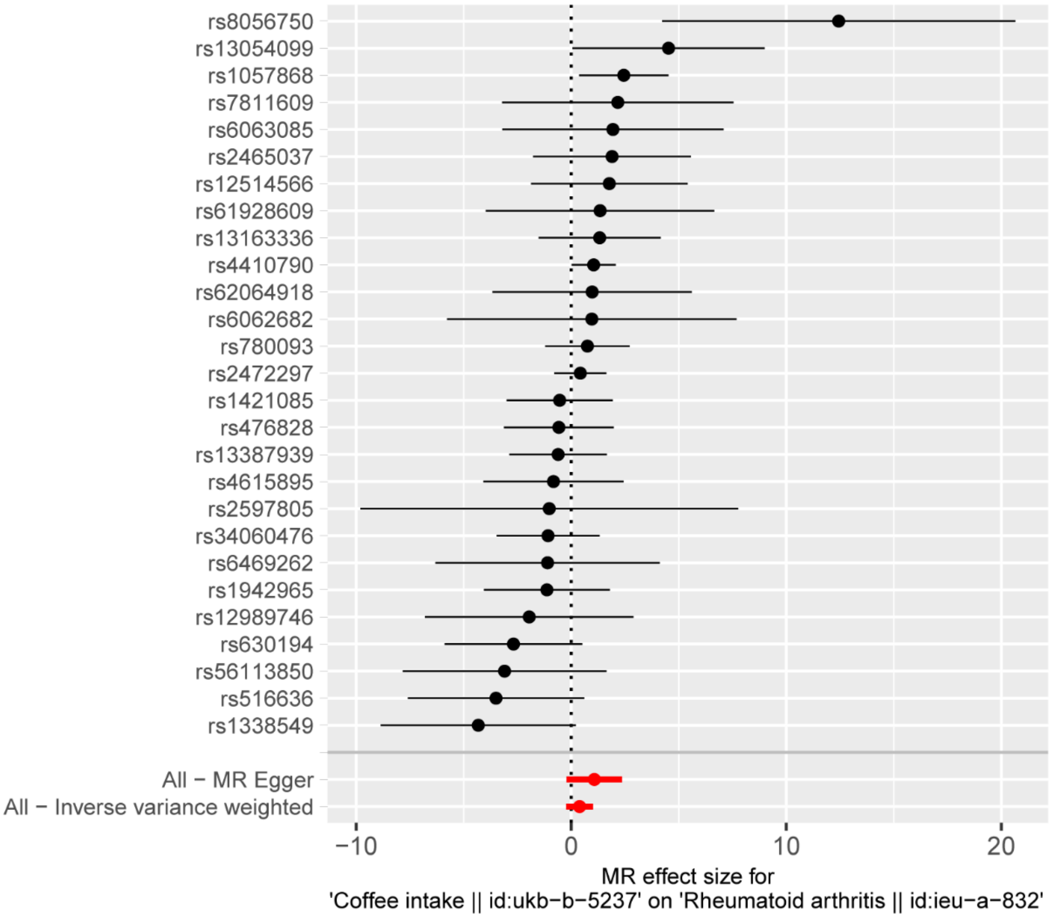

Supplement: Supplementary file 1 [file Data_Sheet_1.PDF]
